# The Impact of Group Drumming on Social-Emotional Behavior in Low-Income Children

**DOI:** 10.1093/ecam/neq072

**Published:** 2011-02-13

**Authors:** Ping Ho, Jennie C. I. Tsao, Lian Bloch, Lonnie K. Zeltzer

**Affiliations:** ^1^Pediatric Pain Program, Department of Pediatrics, David Geffen School of Medicine, University of California, Los Angeles, USA; ^2^Clinical Science Program, Department of Psychology, University of California, Berkeley, CA, USA

## Abstract

Low-income youth experience social-emotional problems linked to chronic stress that are exacerbated by lack of access to care. Drumming is a non-verbal, universal activity that builds upon a collectivistic aspect of diverse cultures and does not bear the stigma of therapy. A pretest-post-test non-equivalent control group design was used to assess the effects of 12 weeks of school counselor-led drumming on social-emotional behavior in two fifth-grade intervention classrooms versus two standard education control classrooms. The weekly intervention integrated rhythmic and group counseling activities to build skills, such as emotion management, focus and listening. The Teacher's Report Form was used to assess each of 101 participants (*n* = 54 experimental, *n* = 47 control, 90% Latino, 53.5% female, mean age 10.5 years, range 10–12 years). There was 100% retention. ANOVA testing showed that intervention classrooms improved significantly compared to the control group in broad-band scales (total problems (*P* < .01), internalizing problems (*P* < .02)), narrow-band syndrome scales (withdrawn/depression (*P* < .02), attention problems (*P* < .01), inattention subscale (*P* < .001)), Diagnostic and Statistical Manual of Mental Disorders-oriented scales (anxiety problems (*P* < .01), attention deficit/hyperactivity problems (*P* < .01), inattention subscale (*P* < .001), oppositional defiant problems (*P* < .03)), and other scales (post-traumatic stress problems (*P* < .01), sluggish cognitive tempo (*P* < .001)). Participation in group drumming led to significant improvements in multiple domains of social-emotional behavior. This sustainable intervention can foster positive youth development and increase student-counselor interaction. These findings underscore the potential value of the arts as a therapeutic tool.

## 1. Introduction

Children under age 18 years represent a quarter of the total population of the USA (74 million) [1]; 39% are low-income, that is, living in families earning less than double the federal poverty level [2]. Although European Americans represent the largest number of low-income children (26%, 10.9 million), other groups are more disproportionately represented: Latino (61%, 9.4 million), African American (60%, 6.5 million), American Indian (57%, 0.3 million), Asian American (30%, 0.9 million), children of immigrant parents (58%, 7.4 million), children of native-born parents (35%, 20.2 million) [2].

### 1.1. Mental Health Needs of Low-Income Youth

Low-income youth are commonly exposed to stressors [3–12] that are well-established risk factors for behavior problems and school failure in the general youth population [13]. Correspondingly, socioeconomic disadvantage is associated with internalizing (e.g., depressive, anxious, somatizing, post-traumatic stress) and externalizing (e.g., antisocial, aggressive, delinquent, substance abusing) behavior in children and adolescents [3, 9, 14–22]. The burden of chronic stress held by low-income youth is compounded by poor access to health and mental health care [1, 22–26]. Moreover, low-income families may be reluctant to obtain services, for reasons ranging from stigma and attitude towards treatment [27, 28] to psychosocial and legal ramifications of reporting problems [12, 29, 30]. Minority youth, in particular, are at greater risk of encountering the “triple threat" of suboptimal health, lack of access to care and inferior services [26].

Notwithstanding, relatively few mental health interventions have targeted low-income youth, and most aim to reduce a single problem behavior or deficit [31–37] in contrast to a positive development approach of increasing core assets that may influence a range of problem behaviors [38, 39]. Positive youth development interventions facilitate positive outcomes through developmentally appropriate achievements intended to address the “whole child" [39].

### 1.2. Group Drumming for Positive Development, Cultural Relevance and Stress Reduction

Group drumming is a recreational music making activity that builds social-emotional assets consistent with a positive youth development approach. It is conducted in a circle and often led by a facilitator whose role is to maximize a sense of community through rhythmic dialogue. Group drumming is inclusive; it is non-verbal, universal, and does not require previous experience for participation. Furthermore, group drumming is culturally relevant; it is an integral part of diverse cultures, and supports the value of collectivism, shared by non-European-based cultures [40].

Previous studies of adults [41–43] and adolescents [44] have shown the biopsychosocial efficacy of group drumming, using protocols involving reflection and self-disclosure to reduce stress. These studies found neuroendocrine and immune changes that were indicative of reduced stress in normal adults with no previous experience in drumming [41], reduced burnout and improved mood in long-term care workers and nurses [42, 43], and improved social-emotional functioning in adolescents from a court-referred residential treatment center [44]. Other art forms used in therapeutic contexts with adults and children have also been linked with improvement in biopsychosocial indicators of stress [45–62].

The unique effectiveness of group drumming with reflection and self-disclosure [41], versus group drumming without these components, suggests the possible added benefit of integrating counseling activities with group drumming to reduce social and emotional manifestations of stress in low-income youth. Social–emotional skill building delivered in a framework of drumming may also confer benefits without the stigma of therapy.

### 1.3. Theoretical Rationale

According to Social Cognitive Theory, group drumming combined with group counseling activities would foster individual self-efficacy and positive outcome expectations through enactive attainment, vicarious experience, verbal persuasion and reduction of physiological arousal [63]. Furthermore, collective efficacy may grow through a shared sense of purpose [63]. In support of this notion, Paulo Freire's Empowerment Education Theory of Dialogue and Praxis asserts that the development of empathy through common experience enables more meaningful reflection and dialogue, which in turn sets the stage for action, or empowerment [64, 65].

### 1.4. Research Objective

In summary, low-income youth are in need of interventions to address social and emotional behavior linked to chronic stress. Therefore, could a school-based group drumming program, integrated with activities from group counseling, improve social and emotional behavior in low-income children?

## 2. Methods

### 2.1. Design and Population

Upon written approval by the Institutional Review Board of the University of California, Los Angeles, as well as the Program Evaluation and Research Branch of the Los Angeles Unified School District (LAUSD), we utilized a pretest-post-test non-equivalent control group design to assess the effects of 12 weeks of school-counselor-led drumming, aimed at social and emotional skill building, on problem behavior in low-income fifth graders. Fifth graders were targeted for early intervention because they would be old enough to benefit from rhythm-based therapeutic activities involving reflection, and their peer-centric developmental stage would lend itself well to group activities [66]. The study was conducted in spring of 2007 at Napa Street Elementary School in the LAUSD. Students at the school were 89% Latino [67], of which *∼*90% were US born [68], 75% were of Mexican ethnicity [68] and 66% were English learners [67]. Ninety-seven percent of the students in the school participated in the reduced-price lunch program, a statewide index of socioeconomic disadvantage [67].

### 2.2. Recruitment

Students from all four fifth-grade classes in the school were recruited for the study. Since there were two classes in each of two different academic scheduling tracks, one class from each track was assigned to the experimental condition, and the other class from each track was assigned to the standard education control condition. Assignment to treatment conditions was not randomized, due to school administrative constraints. The students were told that the study was being conducted to see how drumming might affect their experience in school. They were also told that one group would get weekly drumming for 12 weeks, while the other group would get two sessions after the end of the study.

Of 106 students, 101 obtained parental consent for participation (95%). In one case, non-participation was due to parent refusal. In four cases, consent forms were not returned, and parents could not be contacted. Students that did not have consent to participate went into the control classrooms during the intervention.

### 2.3. Demographics of Participants

Demographic characteristics of the four classes can be found in Table 1. Of 101 participants in the study, 47 (46.5%) were male and 54 (53.5%) were female. Ninety-one (90%) were Latino, five (5%) were African American, two (2%) were Filipino, two (2%) were European American and one (1%) was Asian; these percentages reflected the racial/ethnic demographics of the school [67]. The mean student age was 10.5 years (range 10–12 years). The 10 non-Latino students were spread across all four classrooms. In total, 54 students received the intervention and 47 students were in the standard education control group. There were no significant differences in the proportion of boys versus girls across the four classes. The two experimental group teachers were a European American male, age 43 years, and a Latina, age 33 years; the two control group teachers were European American females, ages 30 and 32 years. There was no loss of student or teacher participation in either the experimental or control groups. 

### 2.4. Building Support from the School Community

Prior to the beginning of the study, all faculty and a few staff from Napa Street Elementary School attended a free in-service drumming workshop at Remo Recreational Music Center in North Hollywood, California. The purpose of this was to increase support for the pilot study from the overall faculty and, in particular, to increase compliance from those that would be involved in the study.

### 2.5. Assessment

The quantitative assessment instrument utilized was the Teacher's Report Form (TRF)—the teacher version of the Child Behavior Checklist—which yields a variety of scales of adaptive functioning derived from a mixed list of 120 factor-loaded items [69]. The TRF asks teachers to rate each of their students on problem behaviors over the past 2 months, with three possible response choices: 0 = not true (as far as you know), 1 = somewhat or sometimes true, 2 = very true or often true. TRF scale scores have been standardized based on normative data from children 6–18 years of age, and the instrument has been subjected to extensive reliability and validity testing [69]. Use of the TRF has been reported in >1000 peer-reviewed publications [70] and its robustness has been demonstrated in multicultural settings as well [71].

The wide range of problem behavior scales offered by the TRF was advantageous, given that the study was exploratory in nature and intended to inform the focus of assessments in future research. The TRF yields three broad-band scales: Total Problems (an aggregate of all items on the rating form), Internalizing Problems (a composite of scores from three narrow-band syndrome scales: Anxious/Depressed, Withdrawn/Depressed, Somatic Complaints) and Externalizing Problems (a composite of scores from two narrow-band syndrome scales: Rule-Breaking Behavior, Aggressive Behavior). The TRF also derives three other narrow-band syndrome scales: Social Problems, Thought Problems, Attention Problems. In addition, it offers two subscales for the syndrome scale of Attention Problems: Inattention and Hyperactivity-Impulsivity. This set of empirically based scales constitutes the core of the TRF.

Items on the TRF can also be used to generate six scales that are consistent with categories from the Diagnostic and Statistical Manual of Mental Disorders, Fourth Edition (DSM-IV), for the American Psychiatric Association [72]: Affective Problems, Anxiety Problems, Somatic Problems, Attention Deficit/Hyperactivity Problems, Oppositional Defiant Problems, and Conduct Problems. Furthermore, the TRF offers two subscales for the DSM-oriented scale of Attention Deficit/Hyperactivity Problems: Inattention and Hyperactivity-Impulsivity. Finally, four other scales can be scored using TRF items: Obsessive-Compulsive Problems, Post-traumatic Stress Problems, Sluggish Cognitive Tempo, and Positive Qualities.

Both experimental and control teachers completed a TRF for each student in her or his class within a 2-week window, immediately prior to the start of the study and immediately upon completion of the study. There was no missing data.

### 2.6. Protocol Development

The group drumming protocol was co-developed by a drum circle facilitator, a public health educator and the school counselor—a licensed clinical social worker familiar with the social, emotional and cultural needs of the research population. The intervention was solely administered by the school counselor and delivered to a whole classroom of students at a time, including the teacher—in order to foster indirect benefits to students through stress reduction, observational learning, and/or carryover of session themes to the classroom. Prior to delivering the intervention, the counselor attended a weekend training session on drum circle facilitation and practiced facilitating drum circles under supervision with five classrooms of students at two socioeconomically similar elementary schools in the LAUSD. This served to confirm the feasibility of working with a whole classroom of students at a time.

To control for the integrity of the rhythmic component of the intervention, the drum circle facilitator, who was experienced in working with children, served as a silent participant observer during the sessions and offered suggestions for improvement during debriefing meetings. A lesson plan was outlined in advance of each session based upon the previous one, and it was delivered with consistency to both classrooms designated to receive the intervention. Upon completion of the study, a scripted manual was developed for future research use.

### 2.7. Intervention

The intervention took place during the school day, right after lunch, for 40–45 min weekly, over a 12-week period. It consisted of a hybrid of activities used in contemporary drum circles and in group counseling with school-age pupils, to maximize development of social and emotional skills through guided interaction, reflection, and self-disclosure. Drums and rhythms were chosen to reflect cultural diversity; drumming activities emphasized process and not performance. Session themes included various combinations of positive behavior, team building, positive risk taking, self-esteem, awareness of others, leadership, sense of self, expressing feelings, managing anger, managing stress, empathy and gratitude. “Focus and listening" was a constant theme.

Each session began with the whole group playing an ongoing rhythm pattern to release stress, energize and establish a sense of community. Focus and listening was encouraged throughout the program via the use of non-verbal cues and “call and response"-type activities, which required the echoing back of an improvised rhythm played by a member of the group. Rhythmic activities were also used as the basis for lessons corresponding with session themes. For example, in the session on positive behavior, participants would simultaneously speak and beat the affirmation, “I am responsible, I do the right thing"; this was followed by a group discussion of the meaning of the affirmation for integration and internalization. In the session on sense of self and awareness of others, children shared their favorite color, food and animal while drumming to the syllables of the words. In the session on expressing feelings and managing anger, the group brainstormed ways to manage anger, learned a spoken “calm down mantra", and then expressed feelings on the drums. In the session on team building and positive risk taking, hand shakers were systematically passed around the circle in increasingly rapid succession until many dropped; this was followed by a discussion of the acceptability of making mistakes in the learning process and how it feels to give and receive. In the session on leadership, empathy and gratitude, when students were given an opportunity to lead a call and response from the center of the circle, other students were asked how the leader may have felt, which segued into a discussion of empathy and gratitude.

Session activities promoted the following constructs found in effective positive youth development programs: social-emotional-cognitive-behavioral-moral competence, self-efficacy, clear standards for behavior, healthy bonding, opportunities for prosocial involvement and recognition, structure and consistency in program delivery [38]. A more complete description of the group drumming protocol and its development will be published in a separate article.

### 2.8. Statistical Analysis

To examine group differences in the current sample, based on changes in scores from baseline to post-intervention/control, difference scores for the TRF scales were calculated by subtracting post-intervention/control scores from baseline scores for all scales. Preliminary analyses to examine the potential effect of the sex of the child on baseline TRF scores did not reveal any differences across the four classes. As the current investigation used a quasi-experimental design and did not randomly assign classrooms to study conditions, the drumming and control classes were not combined. Therefore, a series of ANOVAs were conducted to examine the effect of teacher (TE1 versus TE2 versus TE3 versus TE4) on each of the TRF scales separately. In the event of a significant omnibus *F*, Fisher's least significant difference (LSD) *post-hoc* tests were conducted to specify which groups differed. The distributions of the TRF difference scores were examined to ensure that assumptions for parametric analyses were met. To normalize the distributions, outliers were identified and excluded for individual scales as detailed below. As recommended by Tabachnick and Fidell [73], distributions of scores were examined within each classroom; values >2 SD from the mean were considered outliers. Effect sizes are reported in the form of partial eta squared (*η*_*p*_^2^).

## 3. Results

### 3.1. TRF Broad-Band Scales

Baseline, post-intervention/ control, and difference scores for TRF broad-band scales that were statistically significant are displayed in Table 2. 

**Figure 1:**
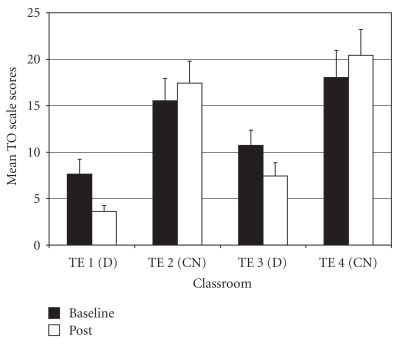
Total problems—mean baseline and post-intervention/control scores by classroom.

*Total Problems* (TO—an aggregate of all items on the rating form). For TO difference scores, there was an overall significant group difference (*F*(3,97) = 5.27, *P* < .01;  *η*_*p*_^2^ = 0.14). *Post-hoc* tests showed that drumming teacher TE1 rated students as improving more on TO scores than the two control teachers (TE2 and TE4) (Figure 1). Also, drumming teacher TE3 rated students as improving more on TO scores than control teacher TE4. 

**Figure 2:**
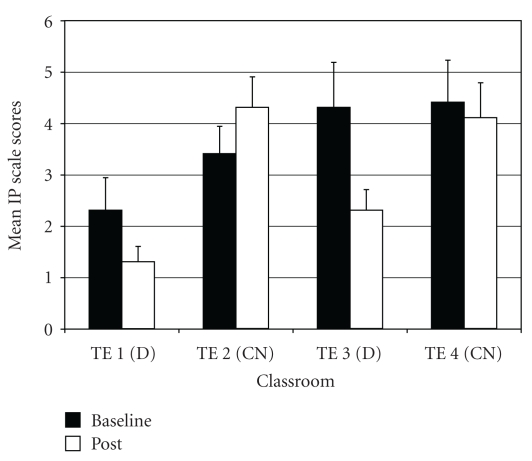
Internalizing problems—mean baseline and post-intervention/control scores by classroom.

*Internalizing Problems* (IP—a composite of scores from three narrow-band syndrome scales: Anxious/Depressed, Withdrawn/Depressed, Somatic Complaints). For IP difference scores, the ANOVA showed a significant difference among groups (*F*(3,97) = 3.73, *P* < .02;  *η*_*p*_^2^ = 0.10). *Post-hoc* tests indicated that drumming teacher TE3 rated students as improving more on IP scores compared to control teacher TE2 (Figure 2). 

*Externalizing Problems* (a composite of scores from two narrow-band syndrome scales: Rule-Breaking Behavior, Aggressive Behavior). Difference scores for externalizing problems were not significant between groups.

### 3.2. TRF Narrow-Band Syndrome Scales

Baseline, post-intervention/control and difference scores for TRF narrow-band syndrome scales that were statistically significant are displayed in Table 3. 

**Figure 3:**
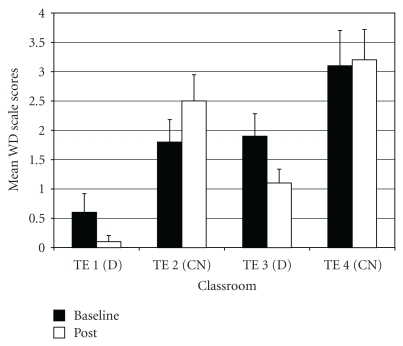
Withdrawn/depression—mean baseline and post-intervention/control scores by classroom.

*Withdrawn/Depressed* (WD). For WD difference scores, two outliers were identified (both in drumming class 1). Exclusion of these outliers normalized the distribution. The ANOVA on WD difference scores showed a significant difference among groups (*F*(3,95) = 3.75, *P* < .02;  *η*_*p*_^2^ =  0.11). *Post-hoc* tests indicated that the two drumming teachers (TE1 and TE3) rated students as improving more on WD scores post-intervention compared to control teacher TE2 (Figure 3). 

**Figure 4:**
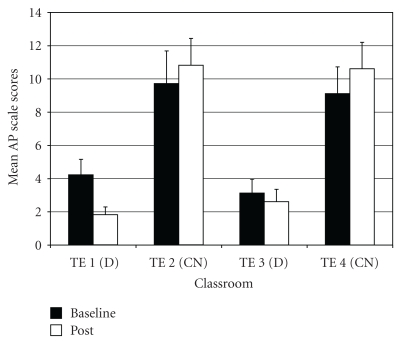
Attention problems—mean baseline and post-intervention/control scores by classroom.

*Attention Problems* (AP). For AP difference scores, results of the ANOVA indicated a significant difference among groups (*F*(3,97) = 5.69, *P* < .01;  *η*_*p*_^2^ = 0.15). *Post hoc* tests showed that drumming teacher TE1 rated students as improving more on AP scores post-intervention relative to the two control teachers (TE2 and TE4) (Figure 4). In addition, drumming teacher TE3 rated students as improving more on AP scores compared to control teacher TE4. 

**Figure 5:**
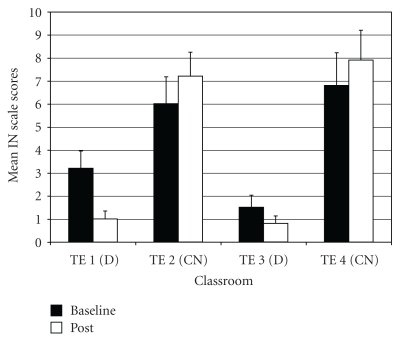
Inattention (subscale of attention problems)—mean baseline and post-intervention/control scores by classroom.

*Inattention* (IN—one of two subscales for Attention Problems; the other being Hyperactivity-Impulsivity). IN difference scores showed a significant difference among groups (*F*(3,97) = 7.16, *P* < .001;  *η*_*p*_^2^ = 0.18). Results of *post-hoc* tests indicated that the two drumming teachers (TE1 and TE3) rated students as improving more on IN scores post-intervention compared to the two control teachers (TE2 and TE4) (Figure 5). 

*Anxious/Depressed, Somatic Complaints, Social Problems, Thought Problems, Rule-Breaking Behavior, Aggressive Behavior, Hyperactivity-Impulsivity Subscale of Attention Problems.* Difference scores for these syndrome scales were not significant between groups.

### 3.3. TRF DSM-Oriented Scales

Baseline, post-intervention/ control, and difference scores for TRF DSM-oriented scales that were statistically significant are displayed in Table 4. 

**Figure 6:**
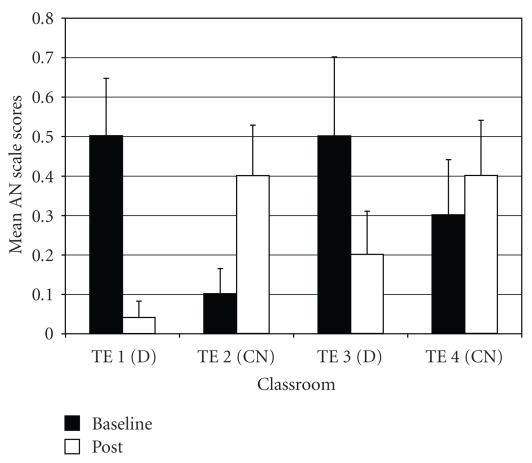
Anxiety problems—mean baseline and post-intervention/control scores by classroom.

*Anxiety Problems* (AN). For AN difference scores, one outlier was identified (in drumming class 1); exclusion of this outlier normalized the distribution of AN scores. Results of the ANOVA on AN difference scores showed a significant difference among groups (*F*(3,96) = 4.97, *P* < .01; *η*_*p*_^2^ =  0.15) *Post-hoc* tests indicated that the two drumming teachers (TE1 and TE3) rated students as improving more on AN scores post-intervention than control teacher TE2 (Figure 6). In addition, drumming teacher TE1 rated students as improving more on AN scores than control teacher TE4.

**Figure 7:**
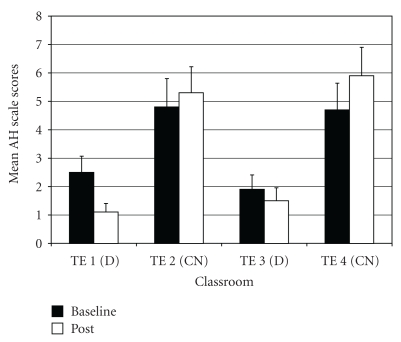
Attention deficit/hyperactivity problems—mean baseline and post-intervention/control scores by classroom.

*Attention Deficit/Hyperactivity Problems* (AH). For AH difference scores, two outliers were identified (both in control class 4), and exclusion of these outliers normalized the distribution. The ANOVA on AH difference scores showed a significant difference among groups (*F*(3,95) = 5.96, *P* < .01;  *η*_*p*_^2^ = 0.14). *Post-hoc* tests indicated that drumming teacher TE1 rated students as improving more on AH scores compared to the two control teachers (TE2 and TE4) (Figure 7). In addition, drumming teacher TE3 rated students as improving more on AH scores relative to control teacher TE4. 

**Figure 8:**
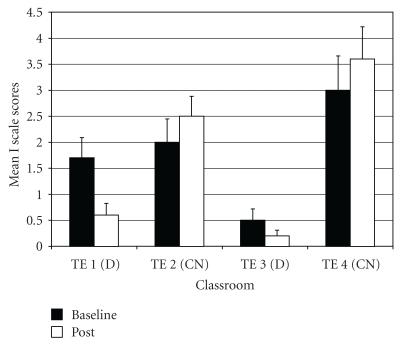
Inattention (subscale of attention deficit/hyperactivity problems)—mean baseline and post-intervention/control scores by classroom.

*Inattention* (I—one of two subscales for Attention Deficit/Hyperactivity Problems; the other being Hyperactivity-Impulsivity). For I difference scores, the ANOVA results showed a significant difference among groups (*F*(3,97)  = 7.51, *P* < .001;  *η*_*p*_^2^ = 0.19). *Post-hoc* tests indicated that drumming teacher TE1 rated students as improving more on I scores than the two control teachers (TE2 and TE4) (Figure 8). 

**Figure 9:**
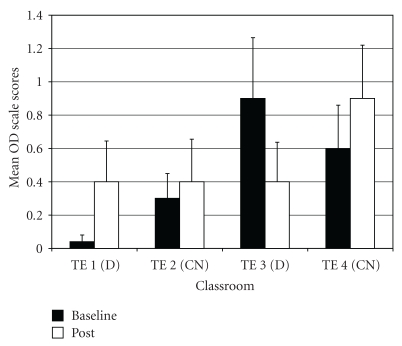
Oppositional defiant problems—mean baseline and post-intervention/control scores by classroom.

*Oppositional Defiant Problems* (OD). For OD difference scores, results of the ANOVA indicated a significant difference among groups (*F*(3,97) = 3.36, *P* < .03;  *η*_*p*_^2^ = 0.09). *Post-hoc* tests showed that drumming teacher TE3 rated students as improving more on OD scores than control teacher TE4 (Figure 9). 

*Affective Problems, Somatic Problems, Conduct Problems, Hyperactivity-Impulsivity Subscale of Attention Deficit/Hyperactivity Problems.* Difference scores for these DSM-oriented scales were not significant between groups.

### 3.4. TRF Other Scales

Baseline, post-intervention/control, and difference scores for TRF other scales that were statistically significant are displayed in Table 5. 

**Figure 10:**
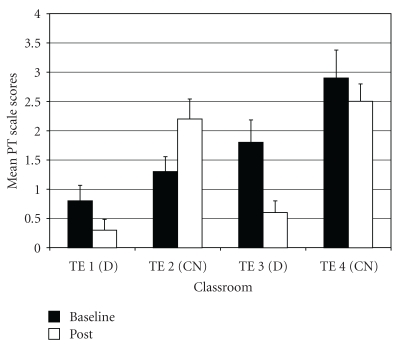
Post-traumatic stress problems—mean baseline and post-intervention/control scores by classroom.

*Post-Traumatic Stress Problems* (PT). For PT difference scores, results of the ANOVA showed a significant difference among groups (*F*(3,97) = 6.40, *P* < .01;  *η*_*p*_^2^ = 0.17). *Post-hoc* tests indicated that drumming teachers TE1 and TE3 rated students as improving more on PT scores compared to control teacher TE2 (Figure 10). In addition, control teacher TE4 rated students as improving more on PT scores compared to the other control teacher TE2. 

**Figure 11:**
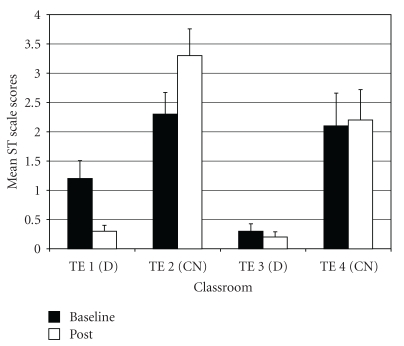
Sluggish cognitive tempo—mean baseline and post-intervention/control scores by classroom.

*Sluggish Cognitive Tempo* (ST). For ST difference scores, one outlier was identified (in control class 2). Exclusion of this outlier normalized the distribution. The ANOVA on ST difference scores showed a significant difference among groups (*F*(3,96)  = 9.29, *P* < .001; *η*_*p*_^2^  = 0.23). *Post-hoc* tests indicated that drumming teacher TE1 rated students as more improved on ST scores compared to the two control teachers (TE2 and TE4), as well as the other drumming teacher TE3 (Figure 11). Also, drumming teacher TE3 rated students as improving more on ST scores than control teacher TE2, and control teacher TE4 rated students as declining less on ST scores than the other control teacher TE2. 

*Obsessive-Compulsive Problems, Positive Qualities.* Difference scores for these other scales were not significant between groups.

## 4. Discussion

This pilot study investigated the impact of group drumming on social-emotional behavior in low-income, primarily Latino, children with the specific aim of identifying the range of behaviors that may show improvement with intervention. The TRF was utilized to assess a myriad of problem behaviors. Students in the school counselor-led drumming program improved significantly compared to the control group in multiple domains of social-emotional behavior. Significant changes were found in broad-band scales (total problems and internalizing problems), narrow-band syndrome scales (withdrawn/depression, attention problems, and inattention subscale), DSM-oriented scales (anxiety problems, attention deficit/hyperactivity problems, inattention subscale, and oppositional defiant problems), and other scales (post-traumatic stress problems and sluggish cognitive tempo). On each of these scales, at least one drumming class did better than at least one control class. These findings support our hypothesis that a school-based group drumming program, integrated with activities from group counseling, would improve social and emotional behavior in low-income children.

### 4.1. Implications

The results of this study suggest that group drumming combined with group counseling may be used effectively to mitigate internalizing problems in a low-income, predominantly Latino, population. This is important not only because Latino youth tend to report more internalizing problems than other youth [74–76], but also because these types of problems are even difficult for their caregivers [77] and physicians [17] to identify. In addition, children manifesting behavior problems in the attention spectrum (including sluggish cognitive tempo, which can be a proxy for inattention) [78] seem to respond well to this intervention.

The effectiveness of the intervention appears to have been due to the combination of drumming and counseling activities, based on surveys of the teacher participants and anecdotal reports by the school counselor, observer, and students involved in the study. In support of this assumption, Bittman et al. evaluated six different protocols (resting control, listening to drumming music, 50% instruction and 50% drumming activity, 20% instruction and 80% drumming activity, facilitated shamanic drumming, and composite drumming involving reflection and self-disclosure facilitated specifically by a music therapist) and found that only the composite protocol led to neuroendocrine and immune changes in adults, at a level indicative of stress reduction [41].

The strength of this intervention was reflected in its effectiveness within a realistic school context [79]. In the name of sustainability, a school counselor was used to deliver the intervention, rather than an expert drum circle facilitator. Moreover, an entire classroom of a mixed composition of up to 30 students was served at a time, despite the fact that a stronger effect may have been achieved by working with groups of 10–15 students at a time [41, 42, 80, 81] or by delivering it only to those screened for critical levels of need [82–86]. In addition, implementation took place during the school day after lunch, which is a difficult time of day to teach based upon an informal survey of elementary school teachers. Finally, study participants were in their spring semester of fifth grade—the most challenging time of year for the most potentially resistant group of elementary school students.

The findings of this study are consistent with other school-based, short-term, group interventions aimed at improving behavior in low-income youth [80–82, 85, 87, 88]. Enhancing school-based services can reduce barriers to mental health care [89, 90], as they are the primary source of such care for youth [90, 91]. School settings are ideally suited for preventive services, extended observation, and coordinated care [89]. School counselor-led group drumming, as conceived in this study, not only expands these possibilities in culturally relevant ways but also offers an additional area in which students can excel. The intrinsic value of drumming, and the opportunity to develop competence in it, may lead to continued participation [92], which may in itself be helpful given the social, emotional and academic benefits that have been linked to participation in music activities [93] and organized activities in general [94]. The National Education Association calls for the use of the arts as a “hook" to get the growing number of Latino students interested in school [95]. School counselor-led group drumming can not only serve as the “hook" but also close an opportunity gap that exists for low-income youth [96, 97].

### 4.2. Limitations

Several methodological issues in this pilot feasibility study need to be considered, and future studies should attempt to address these limitations. First, the effect sizes in the current study (*η*_*p*_^2^ = 0.09–0.23) were small due to the sample size and inclusionary approach to recruitment; however, one cannot underestimate the practical value of a change in behavior of even one student in a classroom [84]. Smaller gains across a broader distribution of risk factors may also have larger public health value [86, 98], particularly given variations in behavioral responses to stress based on race, ethnicity, and gender [18, 99–101]. Furthermore, achieving an effect under challenging circumstances may increase the value of the finding [79]. The effect sizes reported in this study are comparable to those found in meta-analyses of other interventions reported in the literature [36, 86, 102, 103].

Second, random assignment of classrooms to treatment conditions was not feasible due to school administrative constraints; however, selection bias was probably minimized by the homogeneity of the study population. Third, teacher raters were not blinded to the group assignment of the students and, thus, may have been prone to reporting bias; this is a major limitation of the pilot study. Future studies should include cross-informant measures or utilize objective observers in order to corroborate findings. Fourth, the lack of an attention control group [104] and the necessary inclusion of a “gifted" class in the experimental group may have had unintended effects.

**Figure 12:**
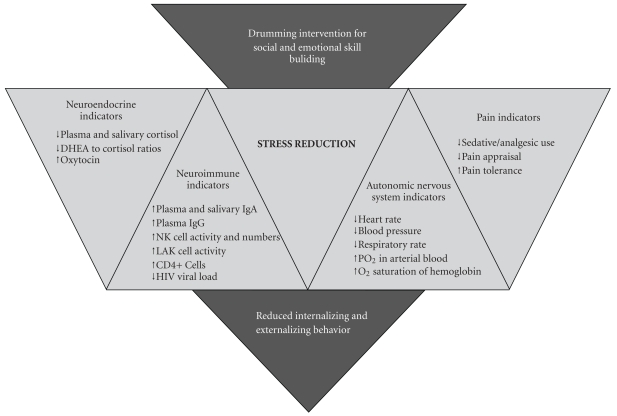
Stress reduction as the proposed mediator between the drumming intervention and reduced internalizing and externalizing behavior.

Finally, this study demonstrated that a group drumming intervention could improve social-emotional indicators of stress; however, in order to identify stress reduction more definitively as the possible mechanism, corresponding biological indicators should be measured.  Figure 12 shows neuroendocrine, neuroimmune, autonomic nervous system, and pain indicators of stress reduction that have been associated with arts-based interventions in the scientific literature [41, 45–47, 49, 51–54, 56–58, 60]. 

### 4.3. Future Research

The results of this study suggest that future investigations linking group drumming and social-emotional behavior in a low-income, primarily Latino, population should focus on assessment of problems in internalizing and attention spectrum domains. However, future studies should also involve larger samples, analyzed for effects on different subgroups of youth by race, ethnicity and gender, as differences in intervention effects have been reported in each of these areas [35, 80, 81, 86, 87]. In order to determine the integrity of results over time, follow up assessments would be necessary. It would also be useful to assess the extent to which a sustained or repeated intervention could mitigate behavior problems—such as substance abuse, gang involvement, and school dropout—that are not typically seen at the elementary school level. Additionally, while this study has demonstrated the effectiveness of group drumming for improving social-emotional behavior in a normative sample, future studies should assess its potential utility in a clinical population. The participants in this study were a non-referred population that showed baseline scores at or below the norm in all behavior scales; therefore, clinical significance cannot be inferred despite the statistically significant reductions in problem behaviors reflected in these scales [4, 9, 18, 69].

Future research should also investigate the effects of the group drumming intervention on academic performance [105, 106], particularly given a meta-analysis of 300+ studies that found academic achievement and behavior significantly improved by social-emotional learning [107]. The study reported here did not utilize the academic performance portion of the TRF, in an effort to reduce the burden on teacher respondents. Additionally, family participation may enhance the effects of the intervention, since family support has been shown to buffer internalizing and externalizing behavior in low-income youth [19, 21, 101, 108–111]. Group drumming lends itself well to family involvement, without the stigma of a mental health intervention [27, 28]. Finally, future studies should evaluate the relative efficacy of intervention delivery by other types of school personnel.

## 5. Conclusions

School counselor-led group drumming, integrated with activities from group counseling, appears to improve the social and emotional correlates of chronic stress in low-income children. Through a positive development approach, the program can increase core assets that may influence a wide spectrum of behaviors, thus yielding broad public health value. This sustainable program can increase student-counselor interaction, provide a feasible alternative to traditional counseling methods that may lose efficacy over time, and serve as a portal to mental health care for those with unmet needs. The results of this study underscore the potential value of the arts as a therapeutic tool.

## Figures and Tables

**Table 1:** Demographic characteristics of the four classrooms.

	TE1 (*n = *24)	TE2 (*n* = 22)	TE3 (*n* = 30)	TE4 (*n* = 25)
Condition	D	CN	D	CN
Sex				
Girls	11 (45.8%)	13 (59.1%)	15 (50.0%)	15 (60.0%)
Boys	13 (54.2%)	9 (40.9%)	15 (50.0%)	10 (40.0%)
Age, mean (SD)	10.58 (0.58)	10.46 (0.67)	10.43 (0.57)	10.48 (0.51)
Race/ethnicity	23 Latino	21 Latino	25 Latino	22 Latino
	1 European American	1 Asian	3 African American	2 African American
			2 Filipino	1 European American

TE: teacher; D: drumming; CN: control.

**Table 2:** Mean (standard deviation) TRF broad-band scale scores by classroom.

	TE1 (*n = *24)	TE2 (*n* = 22)	TE3 (*n* = 30)	TE4 (*n* = 25)
Condition	D	CN	D	CN
TRF scale				
Baseline TO	7.6 (7.9)	15.5 (11.3)	10.7 (9.0)	18.0 (14.6)
Post-TO	3.6 (3.2)	17.4 (11.1)	7.4 (7.9)	20.4 (14.0)
Difference TO	4.0 (7.9)	–1.9 (7.4)	3.3 (6.2)	–2.4 (7.6)
Baseline IP	2.3 (3.1)	3.4 (2.5)	4.3 (4.8)	4.4 (4.1)
Post-IP	1.3 (1.4)	4.3 (2.8)	2.3 (2.2)	4.1 (3.4)
Difference IP	1.0 (3.2)	–0.9 (3.1)	2.0 (3.5)	0.3 (2.6)

TE: teacher; D: drumming; CN: control; TRF: Teacher's Report Form; Difference: difference score (post-intervention/control minus baseline); TO: total problems; IP: internalizing problems. A positive value for difference scores indicates improvement whereas a negative value indicates worsening of symptoms.

**Table 3:** Mean (standard deviation) TRF narrow-band syndrome scale scores by classroom.

	TE1 (*n =* 24)	TE2 (*n* = 22)	TE3 (*n* = 30)	TE4 (*n* = 25)
Condition	D	CN	D	CN
TRF scale				
Baseline WD	0.6 (1.5)	1.8 (1.8)	1.9 (2.1)	3.1 (3.0)
Post-WD	0.1 (0.5)	2.5 (2.1)	1.1 (1.3)	3.2 (2.6)
Difference WD	0.5 (1.6)	–0.7 (2.4)	0.8 (1.3)	–0.1 (1.6)
Baseline AP	4.2 (4.6)	9.7 (9.2)	3.1 (4.6)	9.1 (8.1)
Post-AP	1.8 (2.3)	10.8 (7.6)	2.6 (4.1)	10.6 (8.0)
Difference AP	2.4 (4.0)	–1.1 (4.1)	0.5 (2.3)	–1.5 (4.4)
Baseline IN	3.2 (3.7)	6.0 (5.5)	1.5 (2.9)	6.8 (7.1)
Post-IN	1.0 (1.7)	7.2 (4.9)	0.8 (1.8)	7.9 (6.5)
Difference IN	2.2 (3.1)	–1.2 (3.3)	0.7 (1.5)	–1.1 (3.7)

TE: teacher; D: drumming; CN: control; TRF: Teacher's Report Form; Difference: difference score (post-intervention/control minus baseline); WD: withdrawn/depressed; AP: attention problems; IN: inattention (subscale of AP). A positive value for difference scores indicates improvement whereas a negative value indicates worsening of symptoms.

**Table 4:** Mean (standard deviation) TRF DSM-oriented scale scores by classroom.

	TE1 (*n = *24)	TE2 (*n* = 22)	TE3 (*n* = 30)	TE4 (*n* = 25)
Condition	D	CN	D	CN
TRF scale				
Baseline AN	0.5 (0.7)	0.1 (0.3)	0.5 (1.1)	0.3 (0.7)
Post-AN	0.04 (0.2)	0.4 (0.6)	0.2 (0.6)	0.4 (0.7)
Difference AN	0.46 (0.7)	–0.3 (0.6)	0.3 (0.8)	–0.1 (0.9)
Baseline AH	2.5 (2.8)	4.8 (4.7)	1.9 (2.8)	4.7 (4.5)
Post-AH	1.1 (1.5)	5.3 (4.3)	1.5 (2.5)	5.9 (4.8)
Difference AH	1.4 (2.4)	–0.5 (1.9)	0.4 (1.6)	–1.2 (2.9)
Baseline I	1.7 (1.9)	2.0 (2.1)	0.5 (1.2)	3.0 (3.3)
Post-I	0.6 (1.1)	2.5 (1.8)	0.2 (0.6)	3.6 (3.1)
Difference I	1.1 (1.5)	–0.5 (1.0)	0.3 (0.9)	–0.6 (1.8)
Baseline OD	0.04 (0.2)	0.3 (0.7)	0.9 (2.0)	0.6 (1.3)
Post OD	0.4 (1.2)	0.4 (1.2)	0.4 (1.3)	0.9 (1.6)
Difference OD	–0.36 (0.2)	–0.1 (1.0)	0.5 (1.2)	–0.3 (1.0)

TE: teacher; D: drumming; CN: control; TRF: Teacher's Report Form; Difference: difference score (post-intervention/control minus baseline); AN: anxiety problems; AH: attention deficit/hyperactivity problems; I: inattention (subscale of AH); OD: oppositional defiant problems. A positive value for difference scores indicates improvement whereas a negative value indicates worsening of symptoms.

**Table 5:** Mean (standard deviation) TRF other scale scores by classroom.

	TE1 (*n* = 24)	TE2 (*n* = 22)	TE3 (*n* = 30)	TE4 (*n* = 25)
Condition	D	CN	D	CN
TRF Scale				
Baseline PT	0.8 (1.3)	1.3 (1.2)	1.8 (2.1)	2.9 (2.4)
Post- PT	0.3 (0.9)	2.2 (1.6)	0.6 (1.1)	2.5 (1.5)
Difference PT	0.5 (1.6)	−0.9 (1.8)	1.2 (1.6)	0.4 (1.8)
Baseline ST	1.2 (1.5)	2.3 (1.7)	0.3 (0.7)	2.1 (2.8)
Post-ST	0.3 (0.5)	3.3 (2.1)	0.2 (0.5)	2.2 (2.6)
Difference ST	0.9 (1.4)	−1.0 (1.4)	0.1 (0.3)	−0.1 (1.6)

TE: teacher; D: drumming; CN: control; TRF: Teacher's Report Form; Difference: difference score (post-intervention/control minus baseline); PT: post-traumatic stress problems; ST: sluggish cognitive tempo. A positive value for difference scores indicates improvement whereas a negative value indicates worsening of symptoms.
